# Volumetric modulated arc therapy for thoracic node metastases: a safe and effective treatment for a neglected disease

**DOI:** 10.18632/oncotarget.10826

**Published:** 2016-07-24

**Authors:** Davide Franceschini, Fiorenza De Rose, Antonella Fogliata, Piera Navarria, Anna Maria Ascolese, Ciro Franzese, Tiziana Comito, Angelo Tozzi, Cristina Iftode, Lucia Di Brina, Giuseppe D'Agostino, Elena Clerici, Luca Cozzi, Marta Scorsetti

**Affiliations:** ^1^ Humanitas Cancer Center and Research Hospital, Radiosurgery and Radiotherapy Department, 20089 Milan-Rozzano, Italy; ^2^ Department of Biomedical Sciences, Humanitas University, 20089 Milan-Rozzano, Italy

**Keywords:** mediastinal nodes, SBRT, oligometastases

## Abstract

**Purpose:**

To evaluate the outcome of Stereotactic Body Radiation Therapy (SBRT) with Volumetric Modulated Arc Therapy (VMAT) for thoracic node metastases.

**Results:**

18 out of 29 patients presented with isolated thoracic node metastases with no other sites of disease. Median prescribed dose was 45Gy (range 30–60Gy).

Acute toxicity was recorded as G0 in 28 patients, while one patient was scored as G1. Late toxicity was G0 in 26 patients, one patient was scored G1, one as G2, and one as G4 presented acute myocardial infarction.

During follow up, the best local response was complete remission in 14 patients and partial remission in 11 patients. With a median follow up of 12 months (range 2–35) 9 patients died from disease progression, 10 were still alive with distant metastases, 5 had a locally controlled disease and 5 patients were disease free.

The median OS estimated was 18 months (76%, 49% at one, two years). The median PFS was 9 months (28%, 17% at one, two years).

**Materials and Methods:**

Twenty-nine patients with 32 thoracic nodes metastases were treated with SBRT in our institution. Toxicities and response were assessed. Overall Survival (OS) and Progression Free Survival (PFS) were evaluated.

**Conclusions:**

SBRT is an efficient treatment for thoracic node metastases.

## INTRODUCTION

Thoracic lymph node metastases after curative treatment of primary cancer are a common occurrence during follow up. Incidence of nodal metastases is variable, according to primary histology. Historically, systemic therapies have been the gold standard in such situation. Local treatments, like surgery or radiotherapy (RT), are often limited to a palliative setting, in case of symptomatic disease.

Only recently, local approaches for nodal metastases have been attempted, moving from the promising results of local treatments in oligometastatic patients [1, 2]. According to the first definition of oligometastases by Hellmann and Weichselbaum, proposed in 1995 [3], this state is an intermediate situation, in which a patient presents a limited number of synchronous or metachronous metastases, with the primary tumor either controlled or not. The oligometastatic state is characterized by a slowly progressing disease, in which cancer cells have not yet acquired the features needed for a more diffused and widespread dissemination. According to this theory, the oligometastatic disease can be approached with local treatments, with the objective to impact on disease control and survival and with a non-negligible chance of cure [4].

Stereotactic body radiotherapy (SBRT) is a technique capable of delivering high biologically equivalent dose to the tumor in a small number of fractions with a steep dose fallout on surrounding healthy tissues. If compared with other local therapies, like surgery, SBRT is less invasive and more effective because of decreasing morbidity, less costs and the potential of being delivered on an outpatient basis.

With the improvement of diagnostic technologies, the occurrence of isolated or few nodal metastases is becoming more and more common in clinical practice. Experiences of SBRT in lymph node oligometastases are rare and almost all focusing on abdominal nodes [5, 6, 7].

Indeed, thoracic lymph node metastases are challenging for radiation oncologists, given their proximity to critical structures (e.g. esophagus, great vessels, and trachea). For this reason, surgical removal of these nodes is rarely feasible. Therefore, there is an urgent need for efficient local ablative therapies also for thoracic nodes, like SBRT. Aim of this study is to review our experience in the treatment of metastatic thoracic nodes using Volumetric Modulated Arc Therapy (VMAT) and Flattening Filter Free (FFF) beams. Local control (LC), progression free survival (PFS), overall survival (OS) and toxicity were here analyzed in a cohort of 29 patients.

## RESULTS

### Patient characteristics

Patient characteristics are reported in Table 1. The therapies received by the 29 patients for their primary disease were different and differently combined: surgery, chemotherapy, radiotherapy, since the primary site and histology were different, as shown in the Table.

**Table 1:** Patients characteristics

No. of patients		29
Gender	Male	16
	Female	13
Age at SBRT	Median [y.o.]	67
	Min	24
	Max	84
PS	0	18
	1	10
	2	1
Primary Site	Lung	12
	Breast	4
	Rectum, Colon	6
	Other (pancreas, oesophagus, kidney, thoracic wall)	7
Primary Histology	Adenocarcinoma	16
	Ductal ca.	4
	Squamous cell ca.	3
	Other (clear cell, sarcoma, undiff)	6
Station of mets LN	2	1
	3	3
	4	6
	5	2
	6	2
	7	5
	8	2
	10	11
Chemotherapy (for LN metastases)	yes	9
	no	20
SBRT dose fractionation	5 × 6 Gy	4
	6 × 6 Gy	3
	5 × 8 Gy	1
	6 × 7.5 Gy	11
	6 × 8 Gy	1
	8 × 7.5 Gy	9

The median time from primary tumor diagnosis and mediastinal nodal progression was 29 months (range 0–197 months).

In 14 patients, the thoracic node was the first site of metastatic progression, two patients had stage IV disease from the beginning of their oncological history and nodal metastases were treated because of persisting disease after systemic therapies. Eighteen patients presented with isolated thoracic node metastases with no other sites of disease.

Seven patients did not receive any kind of chemotherapy during their history. In all other cases, a systemic therapy was prescribed at a certain point during patient oncological history (at first diagnosis or at metastases occurrence). Systemic therapies due to thoracic node metastases were prescribed in nine patients, in all other cases SBRT was the only treatment, with medical therapies considered only in case of further progression.

Dose prescriptions and fractionations were reported in Table 1. Only the scheme of 8 fractions of 7.5 Gy delivered more than 100 Gy BED (biological equivalent dose). Median total prescribed dose was 45 Gy (BED 78.8 Gy) with a range of 30–60 Gy (BED 45–105 Gy). Three patients received previous mediastinal irradiation.

### SBRT toxicity

Acute toxicity within 6 months from the SBRT treatment for the mediastinal node was recorded as G0 in 28 patients, while one patient was scored as G1 with asthenia.

Concerning late toxicity, one patient was scored G1 with cough, one as G2 with pneumonia, and one as G4 with acute myocardial infarction. However, this patient had a positive cardiac history and myocardial infarction was probably not related to SBRT. In all other evaluable cases, no late side effect was recorded. Analyzed and reported toxicities are shown in Table 2.

**Table 2:** Acute and late toxicities

ACUTE TOXICITIES	ANY GRADE	G0	G1	G2	G3	G4	G5
Pneumonitis	0	0	0	0	0	0	0
Chest Pain	0	0	0	0	0	0	0
Cough	0	0	0	0	0	0	0
Dyspnea	0	0	0	0	0	0	0
Asthenia	1	0	1	0	0	0	0
Esophagitis	0	0	0	0	0	0	0

LATE TOXICITIES	ANY GRADE	G0	G1	G2	G3	G4	G5
Pneumonitis	1	0	0	1	0	0	0
Cough	1	0	1	0	0	0	0
Dyspnea	0	0	0	0	0	0	0
Esophagitis	0	0	0	0	0	0	0
Chest pain	0	0	0	0	0	0	0
Fistula	0	0	0	0	0	0	0
Cardiac toxicity	1	0	0	0	0	1	0

### Local response

**Figure 1:**
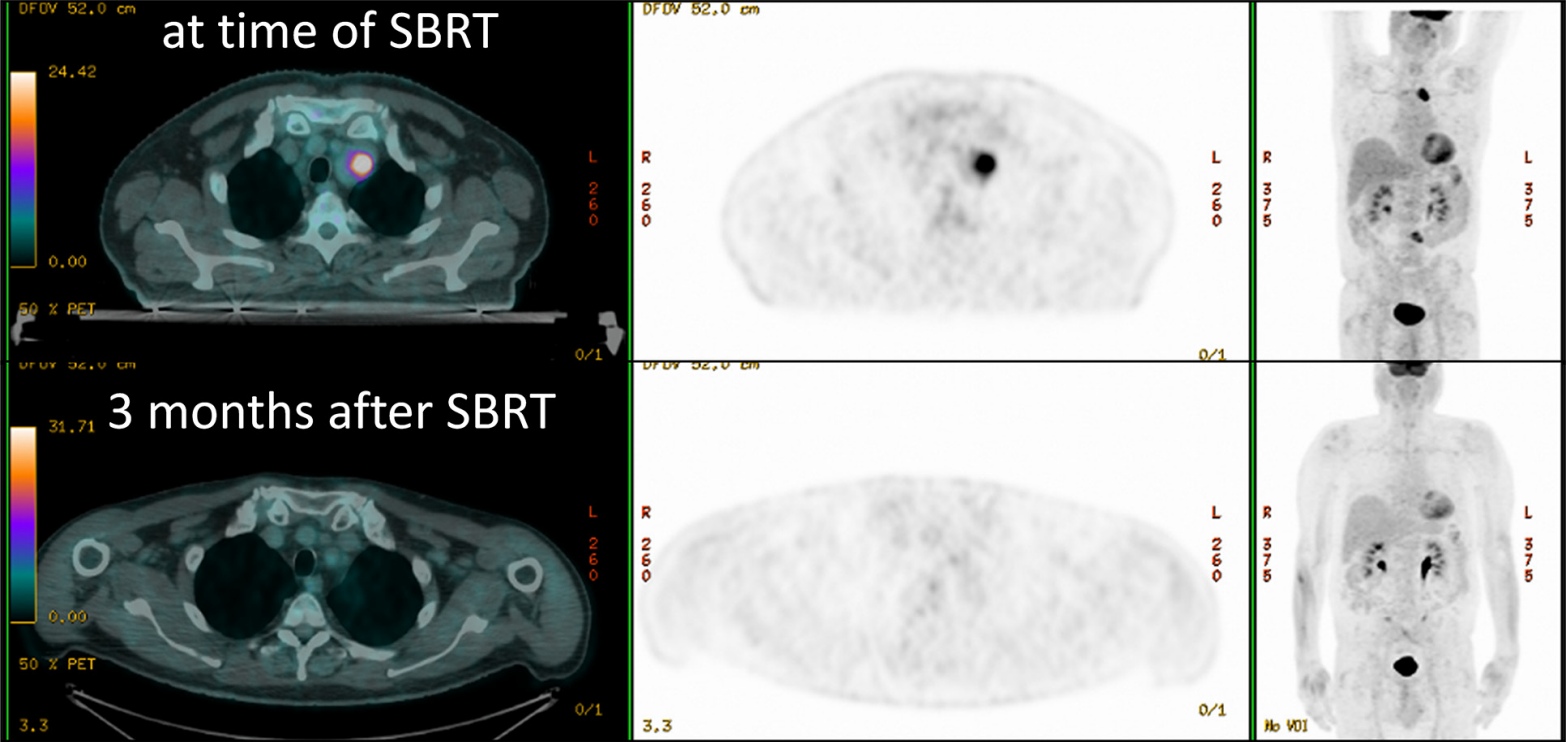
Patient treated with SBRT for a nodal metastases (dose distributions in Figure 3), with complete metabolic response 3 months after treatment

During follow up, the best local response was complete remission in 14 patients and partial remission in 11 patients. In 3 cases a stable disease was found, while one patient presented progressive disease within 4 months from the SBRT treatment. Local progression was diagnosed during follow up in other 3 cases, all other patients maintained local control during follow up. Seventeen patients had a further distant progression during follow up. An example of complete response after SBRT is shown in Figure 1.

### Survivals

With a median follow up of 12 months (range 2–35) nine patients died from disease progression, ten patients were alive at last follow up visit with distant metastases and five patients had a locally controlled disease. At last visit, five patients were still disease free.

The median OS estimated on the whole patient cohort was 18 months (76% at one year, 49% at two years), with a median follow-up time of 12 months (range 2–35). The median PFS was 9 months (28% at one year, 17% at two years).

**Figure 2:**
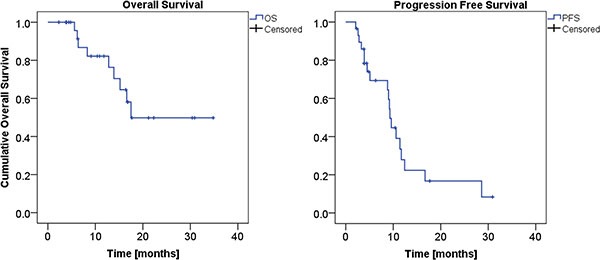
Overall Survival (left panel), and Progression Free Survival (right panel) KM curves

Estimated mean OS for patients presenting complete response after SBRT was 28 months (the median was not reached), to compare with the 14 months of the patients not in complete remission (*p* = 0.050). Estimated median PFS were 11 and 9 months respectively for patients in complete remission or not (*p* = 0.21). OS and PFS in the whole series are shown in Figure 2.

OS relative to patient age at SBRT for thoracic node treatment presents a more favorable trend (not significant, *p* = 0.37) for elderly patients with a mean estimated survival of 19 months for patients younger than 67 years of age (median age of the cohort), to compare with the 27 months for older patients. This trend is significant (*p* = 0.03) for PFS, with a mean (median) estimated survival of 8 (9) months and 16 (12) months for younger and older patients, respectively.

Predictor for OS was the site of the primary disease, *p* = 0.001 (while not significant, *p* = 0.40, for PFS): the median OS for primary breast tumor was 6 months, for colorectal cancer it was 14 months, for primary lung cancer it was 18 months, while for the other primary sites together (including pancreas, esophagus, kidney, soft tissues) the median OS was not reached by the cohort, having an estimated mean OS of 30 months.

Similarly, predictor for OS (*p* = 0.001, while for PSF there is no significance, *p* = 0.11) was the histology of the primary disease: the median OS for ductal carcinoma was 5 months, for adenocarcinoma 16 months, for squamous cell carcinoma the median OS was not reached, having an estimated mean OS of 29 months.

Gender was found to be statistically a predictor for both OS (*p* = 0.015) and PFS (*p* = 0.04), with male having better prognosis: mean estimated OS was 12 and 28 months and PFS was 7 and 14 months for female and male, respectively.

No trend in OS or PFS concerning the time from the prior progression (before the mediastinal nodal progression) and the SBRT was seen.

The dose prescription in terms of BED was not a predictor for OS or PFS (*p* > 0.30 for a cutoff of 100 Gy BED), nor the administration of the chemotherapy after SBRT.

As expected, the OS was significantly better for patients not presenting other sites of metastases (*p* = 0.002), with a mean estimated survival rate of 13 months for patients with other metastatic disease, to compare with the 31 months for patients without other metastases (the median time was not reached in this last group). Also the PFS is significant (*p* = 0.04) with 7 (5) and 14 (11) months as mean (median) estimated survivals, respectively for patients with and without other metastatic location.

Interestingly, patients who received chemotherapy for the mediastinal nodal disease had a worse OS (*p* = 0.003), with a mean estimated survival of 12 months for patients receiving chemotherapy, to compare with the 28 months for patients not receiving drugs – the median time was not reached in this last group. The PFS tended to be significant (*p* = 0.08) with 6 (9) and 13 (11) months as mean (median) estimated survivals, respectively for patients whom the chemotherapy was or was not administered.

## DISCUSSION

Despite a not negligible prevalence, treatment for metastases in thoracic nodes is still unclear and unsatisfactory. Surgical removal is often impossible and RT is commonly delivered with palliative doses, like 30 Gy in 10 fractions. Systemic therapies are the gold standard, but they are loaded with significant toxicity and often poor results in terms of prolonged disease control.

Thoracic node metastases after curative treatment of primary cancer are considered a sign of disease dissemination and treated often in a palliative way. However, in some cases, these metastases can be expression of an oligometastatic, slowly progressive disease.

In these particular situations, local treatments like SBRT are, in our opinion, particularly attractive, being less toxic than systemic drugs and potentially able to obtain a long term disease remission or control. Nowadays, SBRT for lung or liver oligometastases is a well-known and used therapeutic option in selected patients, with excellent results in terms of local control [8]. Experience with nodal metastases is more limited, unfortunately. Although still heterogeneous and sparse, there are data in literature showing control rates higher than 60% achieved with minimal toxicity [9]. Almost all these data are collected in the setting of abdominal nodes, being thoracic nodes still an unexplored setting. To our knowledge, there is only one published series of thoracic nodes treated with SBRT. Meng et al. recently published their retrospective experience on NSCLC with recurrent or second primary mediastinal lymph node metastases [10]. They treated 33 patients with 36 metastatic nodes with SBRT, with doses ranging from 24 to 60 Gy in 3 to 15 fractions. With a median follow up of 20.9 months, authors reported a median OS of 25.5 months; with 1-year, 3-year, and 5-year OS rates of 72.7%, 40.7%, and 20.4%, respectively, with better survival in the subgroup of patients not previously irradiated on mediastinum and in patients with a disease free interval longer than 15.5 months. The addiction of chemotherapy to SBRT gave a small advantage, not statistically significant. The 1-year and 3-year actuarial LC rates for all eligible patients were 100% and 86%, respectively. Authors also reported a significant (> G3) acute and late toxicity in 3 patients and 4 patients respectively, with two treatment related deaths, both in patients treated for subcarinal metastases and already irradiated in the same region.

A direct comparison between our study and the experience published by Meng et al. is obviously difficult, as only 12 patients were affected by primary lung cancer. Differently from them, in our study we did not report a significant toxicity, probably because only three patients in our series had already received previous mediastinal irradiation. We did not observe any toxicity in excess in these three patients neither in patients with subcarinal metastases. Survival reported in our study are inferior, but this can be simply explained by a different patient selection. In Meng's study, patients had a locoregional recurrence of lung cancer, while in our experience more than half of the patients had nodal metastases as a result of metastatic dissemination. Many patients in our series had also received previous chemotherapy. Breast cancer patients, in particular, were heavily pretreated with systemic therapies, when referred to our attention for SBRT.

As in any case of SBRT for oligometastatic disease, selection of ideal candidate is crucial. Although strongly limited by the small number of enrolled patients and by its retrospective nature, our study can give some interesting indications that must be confirmed in future prospective trials.

Elderly patients had a trend towards a better OS in our experience. This result is probably a combination of different factors. Elderly patients could probably have a more indolent disease. Moreover, elderly patients are often under-treated because of comorbidities, performance status or simply because of age and concerns about side effects. Therefore, elderly patients referred for SBRT are not heavily pretreated with systemic therapies and they are often referred for SBRT early in their oncologic history, due to the lack of other therapeutic options. On the contrary, SBRT is well tolerated also in elderly patients as confirmed in previously published experiences [11]. Considering that the World Health Organization estimated an increase in the total number of new cancer patients of about 25% for 2030 in Europe, and this increase is expected to be predominantly (91%) observed in patients aged 65 years or above [12], it deserves to be highlighted that SBRT in elderly patients is safe and efficient and should be considered more and more often.

Breast cancer patients had the worse prognosis in our series, followed by colorectal cancer patients. This is a quite unexpected result, because usually breast cancer patients are regarded as the patients with the best prognosis in studies on metastatic disease. Also in the setting of oligometastatic patients treated with local approaches, breast cancer has the best results [13]. By trying to explain this discrepancy, we observed that breast cancer patients in our series have the longest time from the first diagnosis to SBRT. Although disease free interval (DFI) did not have a significant impact on outcomes in our experience, it is possible that patients with breast cancer, which is a chemo-sensitive disease, often arrive “late” to the radiation oncologist, after being treated with many lines of medical therapies. This could reflect in a more advanced stage of disease, maybe not a real oligometastatic state, being widespread dissemination disguised by previous medical therapies.

We also observed that receiving systemic therapies because of the nodal progression before SBRT is correlated with a worse prognosis. This is partly related to what was previously stated regarding breast cancer, i.e. a late referral for SBRT. Moreover, patients candidate to medical therapies often presented with more metastatic sites, beyond the nodal lesion. In the experience by Meng et al [10], the OS showed a slight trend towards superiority of SBRT with chemo over SBRT without chemo, although these differences were not statistically significant (*p* = 0.35).

We are not saying that young female patients with breast cancer metastases should not be considered for local approaches in the case of oligometastatic disease. On the other hand, we do not want to underestimate the benefits of systemic therapies. What we can suggest moving from our experience is that the timing of local approaches is probably crucial. Therefore, a multidisciplinary discussion on oligometastatic patients should be encouraged in a very early stage, possibly before the beginning of any systemic therapy. Chances of local control, longer overall survival and maybe cure are significantly higher if local approaches are attempted when disease is very limited. A multidisciplinary approach is indispensable. Indeed, as we already know from literature, a combination of local approaches and systemic therapies gives the best outcomes in stage IV breast cancer patients [14–16].

Some of the predictive factors identified in our experience are easy to understand, for instance, the absence of other metastatic sites. On the contrary, others are difficult to understand.

Male patients have a better prognosis in our experience, also if breast cancer patients are excluded from analysis. A clinical explanation of this result is difficult; no clear differences in terms of age or performance status between male and female patients came out from our experience. The small number of patients is likely the easiest explanation, however, we will conduct a prospective trial, trying also to confirm or disprove this observation.

Concerning histology, squamous cell carcinomas had a better outcome in our patients. However, only three patients had a squamous carcinoma, so this result must be confirmed in a prospective way with a larger number of patients.

Meng et al. reported a significant impact of DFI on OS [10]. In their experience, patients who received SBRT < 15.5 months after their surgery, the median OS was 42.0 months vs. 72.0 months for those treated at a ≥ 15.5 months interval (*p* = 0.03). However, DFI did not have a significant impact on LC, PFS or OS in our study. In the same way, we did not find any difference between patients who were already irradiated on mediastinum and patients who were RT-naive. However, only three patients received previous RT in our series, therefore a statistically significant difference cannot emerge. As predictable, with larger numbers, Meng et al. showed that patients who received prior RT had a shorter median OS compared to those without RT (15.3 months vs. 45 months).

We are aware of the limits of the present study, first of all the small number of patients, the heterogeneity of treatment received and the retrospective nature. Moreover, patients in our series are significantly heterogeneous in terms of histology and burden of disease at the time of SBRT. These limits, unfortunately, strongly limit the level of evidence of data emerging from the present series. For this reason, we are currently running a phase II prospective trial that will luckily generate stronger evidences. However, we think that this retrospective study can already give a new prospective in a previously ignored setting. Patients with thoracic node metastases strongly need more efficient and targeted treatment, apart from systemic therapies. SBRT is the perfect weapon for these cases, being not only safe and feasible, as no toxicity alert emerged from our experience, but also effective and promising. It can already guarantee a good local control and avoid the occurrence of severe symptoms in case of disease progression. Furthermore, if we will be able to identify the right candidate for these local approaches, SBRT could have also a significant impact on DFI, OS and a not negligible chance of cure in a metastatic patient.

## MATERIALS AND METHODS

### Patient selection

Between January 2012 and September 2015, 29 patients with 32 thoracic nodes metastases were treated with SBRT in our institution. Their data were retrospectively collected and their analysis was approved by the Humanitas Cancer Center Ethical Committee. All patients were treated in agreement with the Helsinki declaration.

### Thoracic nodes planning and treatment

**Figure 3:**
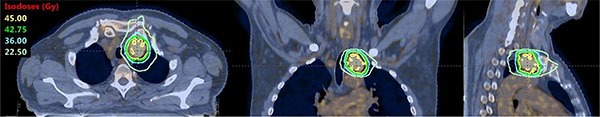
Example of dose distribution of a SBRT plan in a patient presenting primary lung tumor, two nodal metastases, para-aortic and thoracic Here the thoracic treatment plan of 45 Gy in 6 fractions.

All patients underwent contrast-enhanced 4 DCT scan and FDG-CT PET for target definition and treatment preparation. All patients were immobilized in supine position with thermoplastic masks. No respiratory gating nor breath control systems were used. Target and critical structures were outlined per each individual patient. The clinical target volume (CTV) included the metastatic lymph node and was delineated as ITV (internal target volume) on the 4DCT images. A margin of 5 mm was added to CTV in all directions to generate planning target volume (PTV). In all cases, critical structures were: lungs, oesophagus/stomach, heart, large vessels, main bronchus/trachea and spinal cord. Dose objectives for these organs are shown in Table 3. The mean (and standard deviation of the mean) PTV volume over the whole patient cohort was 37.1 ± 5.7 cm^3^. SBRT treatments were delivered with VMAT technology in its RapidArc form, on one of our Varian TrueBeam linear accelerators equipped with either Millennium 120-MLC (5 mm leaf width) or HD-120 MLC (2.5 mm leaf width); the beam energy was 10 MV with FFF (flattening filter free) mode, allowing a maximum dose rate of 2400 MU/min. The arc arrangement was with two partial arcs (160 to 220 degree each) avoiding the entrance through the contralateral side to keep the dose bath as low as possible. Treatment planning was performed on Varian Eclipse system version 11: optimization process used the Progressive Resolution Optimizer algorithm (PRO3, version 11), and the dose distribution was computed with the AAA (Anisotropic Analytical Algorithm, version 11) photon dose calculation algorithm, with a calculation grid of 1.5 mm. An example of dose distribution is shown in Figure 3.

**Table 3:** Dose objectives for organs at risk and target coverage

	6–8 fractions	5 fractions
PTV	V_95%_ > 95%	V_95%_ > 95%
	V_107%_ < 3%	V_107%_ < 3%
	D_max_ < 110%	D_max_ < 110%
Combined Lungs (– CTV)	V_5 Gy_ < 30%	V_5 Gy_ ≤ 30%
	V_10 Gy_ < 20%	V_10 Gy_ ≤ 17%
	V_20 Gy_ < 10%	V_20 Gy_ ≤ 12%
	D_Mean_ ≤ 4 Gy	V_30 Gy_ ≤ 7%
Spinal cord		V_22.5 Gy_ < 0.25 cm^3^
	D_1 cm3_< 32 Gy	V_13.5 Gy_ < 0.5 cm^3^
		D_max_ < 30 Gy
Oesophagus/stomach		D_max_ < 105%
	D_1 cm3_ < 40 Gy	D_1cm3_ < 30 Gy
	D_max_ < 105%	D_5 cm3_ < 27.5 Gy
Heart		D_max_ < 105%
	D_1 cm3_ < 44 Gy	D_1 cm3_ < 40 Gy
	D_15 cm3_ < 32 Gy	D_5 cm3_ < 20 Gy
		D_15 cm3_ < 32 Gy
Large vessels		D_max_ < 105%
	D_1 cm3_ < 105% (63 Gy)	D_10 cm3_ < 47 Gy
	D_1 cm3_ ≤ 40 Gy	D_10 cm3_ < 47 Gy
Main bronchus/trachea		D_max_ < 105%
	D_1 cm3_ < 44 Gy	D_4 cm3_ < 18 Gy
	D_4 cm3_ < 18 Gy	V_35 Gy_ ≤ 1 cm^3^

Patients were treated with SBRT with different fractionation scheme according to nodal site and number, organs at risk proximity and previous mediastinal irradiation. Prescribed schedules were 45 Gy in 6 fractions, 48 Gy in 6 fractions, 60 Gy in 8 fractions, 30 Gy in 5 fractions, 36 Gy in 6 fractions and 40 Gy in 5 fractions.

Treatment image guidance to ensure accurate patient positioning was performed by means of cone beam CT (CBCT) at every session. Patients were evaluated for toxicity halfway during treatment and on the last day of SBRT.

Patient evaluation included history and physical examination, blood tests (including a metabolic panel and hematological profile), KPS, and toxicity assessment. Hematologic and non-hematologic toxicities were graded according to Common Terminology Criteria for Adverse Events version 4.0.

Contrast-enhanced CT scan imaging was performed within 2 months after RT and every 3 months thereafter. FDG-CT PET was performed at 9 months after RT and/or in case of suspected persisting disease.

Response assessment as local control was evaluated using the RECIST criteria (version 1.1) as follows:

Complete Response (CR): disappearance of all target lesions; Partial Response (PR): at least a 30% decrease in the sum of diameters of target lesions, taking as reference the baseline sum diameters; Progressive Disease (PD): at least a 20% increase in the sum of diameters of target lesions, taking as reference the smallest sum on study (this includes the baseline sum if that is the smallest on study); in addition to the relative increase of 20%, the sum must also demonstrate an absolute increase of at least 5 mm; Stable Disease (SD): neither sufficient shrinkage to qualify for PR nor sufficient increase to qualify for PD, taking as reference the smallest sum diameters while on study.

### Data analysis

Primary endpoint of this analysis was local control, defined as the best local response, according to the above RECIST criteria, obtained during follow up. Secondary endpoints were Overall Survival (OS), Progression Free Survival (PFS) and toxicity. OS was defined as the time between the date of the SBRT and the date of death or the date of the last follow-up for censored patients. PFS was defined as the time between the date of the SBRT and the date of first event (local progression of the irradiated node(s) or distant progression of new or previously stable metastatic sites) or the last follow up for censored patients still negative for relapse. Toxicity was defined acute when occurring up to six months after SBRT, late toxicities occurred 6 months after SBRT.

OS and PFS were evaluated with the Kaplan-Meier curves, and Log-Rank tests were analyzed to compare data groups. Due to the small group of selected patients, only univariate analysis was performed for various parameters to evaluate possible predictors. The significance level was considered at 0.05.

## CONCLUSIONS

SBRT is a safe and efficient treatment for thoracic nodes metastases in oligometastatic patients. Selection of patients that can benefit from local ablative treatments is crucial.
